# Single-Cell Transcriptome Analysis of Acute Myeloid Leukemia Cells Using Methanol Fixation and Cryopreservation

**DOI:** 10.3390/diseases12010001

**Published:** 2023-12-20

**Authors:** Lamia Madaci, Charlyne Gard, Sébastien Nin, Alexandre Sarrabay, Céline Baier, Geoffroy Venton, Pascal Rihet, Denis Puthier, Béatrice Loriod, Régis Costello

**Affiliations:** 1 TAGC, TGML, INSERM, UMR1090, Aix-Marseille University, Parc Scientifique de Luminy, 13009 Marseille, France; lamia.madaci@inserm.fr (L.M.); charlyne.gard@inserm.fr (C.G.); sebastien.nin@inserm.fr (S.N.); geoffroy.venton@ap-hm.fr (G.V.); pascal.rihet@univ-amu.fr (P.R.); denis.puthier@univ-amu.fr (D.P.); beatrice.loriod@inserm.fr (B.L.); 2 Advanced BioDesign, Parc Technologique de Lyon, 655 Allée des Parcs, 69800 Saint Priest, France; 3 Hematology and Cellular Therapy Department, Conception University Hospital, 13005 Marseille, France

**Keywords:** single-cell RNA sequencing, acute myeloid leukemia, methanol fixation, rehydration, cryopreservation, transcriptome

## Abstract

Introduction: The application of single-cell RNA sequencing has greatly improved our understanding of various cellular and molecular mechanisms involved in physiological and pathophysiological processes. However, obtaining living cells for this technique can be difficult under certain conditions. To solve this problem, the methanol fixation method appeared as a promising alternative for routine clinical use. Materials and Methods: In this study, we selected two AML samples that had been fixed in methanol for 12–18 months. Once the cells were rehydrated, these samples were subjected to single-cell RNA sequencing. We then compared the results obtained from these samples with those obtained from the same samples cryopreserved in DMSO. Results: We used a previously validated methanol fixation protocol to perform scRNA-seq on DMSO cryopreserved cells and cells fixed in methanol for more than one year. Preliminary results show that methanol fixation induces some genetic and transcriptional modification compared with DMSO cryopreservation but remains a valuable method for single-cell analysis of primary human leukemia cells. Conclusions: The initial findings from this study highlight certain resemblances in methanol fixation over a 12-month period and cryopreservation with DMSO, along with associated transcriptional level modifications. However, we observed genetic degradation in the fixation condition when extending beyond one year. Despite certain study limitations, it is evident that short-term methanol fixation can be effectively used for leukemia blast samples. Its ease of implementation holds the potential to simplify the integration of this technique into routine clinical practice.

## 1. Introduction

The advent of next-generation sequencing has brought about a revolutionary transformation in the realm of scientific and medical research. This breakthrough technology has granted us unprecedented insights into the intricate mechanisms underlying the development of diverse cancer types, clonal evolution, resistance mechanisms against treatments, and the evasion of tumors from the immune system [1,2]. 

Over the past two decades, the advancement of high-throughput technology has facilitated the sequencing of RNA at the individual cellular level, commonly referred to as single-cell RNA sequencing (scRNA-seq).

Single-cell RNA sequencing (scRNA-seq) is an invaluable tool that enables the examination of complex cellular transcriptomes at a single-cell level within biological systems characterized by cellular heterogeneity [3,4]. By combining high-throughput sequencing with advanced bioinformatics tools, scRNA-seq enables the analysis of gene expression across various cell types within each tissue, facilitating the identification of rare and highly diverse cell populations [5]. This technique has found widespread application in numerous fields such as embryogenesis, developmental biology, immunity, neurology, and particularly oncology, where it allows for the simultaneous analysis of tumor cells and their microenvironment [6,7,8]. In a previous publication, we discussed the utility of single-cell analysis in acute myeloid leukemia (AML), as it provides a more informative perspective on chemotherapy resistance by examining the co-expression of oncogenes/anti-oncogenes and mutations at the individual cell level, surpassing the limitations of bulk analysis [3].

The various protocols for single-cell RNA sequencing necessitate the use of living cells due to the rapid transcriptional program modifications that occur upon separation from whole blood. Cryopreservation has emerged as a widely used method for cell preservation, as it provides convenient access to cells at any time and reduces variability in multicenter assays [9]. However, cryopreservation comes with certain limitations. To ensure optimal cell viability for sequencing, cells need to be frozen for a maximum of three weeks at temperatures ranging from −80 °C to −150 °C or frozen in liquid nitrogen for improved viability yields [10]. Additionally, cryopreservation and thawing processes induce the production of specific molecules and cytokines, as well as trigger cellular processes like apoptosis and necrosis, which are not observed in fresh peripheral blood mononuclear cells (PBMCs) [11]. Alternatively, cell fixation has emerged as an alternative solution, commonly used to preserve samples for postmortem analysis or tissue preservation to prevent degradation of tissues and their constituents. Moreover, it serves as a safe procedure for analyzing cells from patients infected with pathogens such as HIV viruses [12,13]. Fixation effectively halts the changes and disruptions induced by freezing and thawing processes. Among the different fixation techniques, methanol fixation appears to be highly efficient and induces minimal or no transcriptomic modifications [14]. Alles et al. developed a simple protocol for cell fixation using methanol, wherein cells were fixed with a cold 80% pure methanol solution and subsequently stored at −80 °C [15]. This method, originally used for Drosophila cells, was adapted for PBMCs by Wang et al. in 2021 [16].

Given the paramount importance of single-cell analysis in research advancements and the development of novel therapeutic strategies [3,17], we conducted a feasibility study to evaluate the suitability of the methanol fixation technique for scRNA-seq analysis of acute myeloid leukemia (AML) cells.

## 2. Materials and Methods

### 2.1. Patients, Samples Preparation, and Methanol Fixation

For this study, Human Peripheral Blood Mononuclear Cells (PBMCs) were collected from consented patients diagnosed with acute myeloid leukemia (AML) (Table 1). The isolation of PBMCs from peripheral blood involved a centrifugation process to separate them from plasma and other components of peripheral blood. Ficoll–Hystopaque density gradient centrifugation, as described by English and Andersen [18], was adapted for this purpose.

Following the isolation of PBMCs from whole blood, two preservation methods were used: cryopreservation and methanol fixation. Cryopreservation involved freezing the cells at either −80 °C or in liquid nitrogen at −195.79 °C for an extended duration. To facilitate this process, PBMCs were stored in a freezing medium containing 90% FBS and 10% DMSO.

The methanol fixation and rehydration procedures were performed following the demonstrated protocol “Methanol Fixation of Cells for Single Cell RNA Sequencing” with 10X Genomics (CG000136, Rev E). PBMCs, ranging from 1 × 10^6^ to 2 × 10^6^, were centrifuged at 300× *g* for 5 min at 4 °C. The resulting cell pellet was resuspended in 200 µL of PBS containing 0.04% Bovine Serum Albumin (BSA) or in cold 1x DPBS. Following this, the cells were fixed by adding 800 µL of pre-chilled 100% methanol in a dropwise manner to prevent cell clumping, and the cell suspension was gently mixed. Subsequently, the methanol-fixed PBMCs were incubated at −20 °C for 30 min and stored at −80 °C for several months.

For the rehydration process, the methanol-fixed PBMCs were transferred from −80 °C to 4 °C and incubated for 5 min, followed by centrifugation at 1000× *g* for 5 min. The methanol was carefully removed, and the resulting cell pellet was resuspended in rehydration buffer, which comprised 3x SSC buffer, 0.04% BSA, 1 mM DTT, and 0.2 U/µL RNase inhibitor. To eliminate any debris and cell clumps present in the suspension after the washes, the PBMCs were passed through a 40 µm Flowmi cell strainer. Subsequently, the cell concentration was determined by counting the cells using the LUNA FL cell counter.

**Figure 1:**
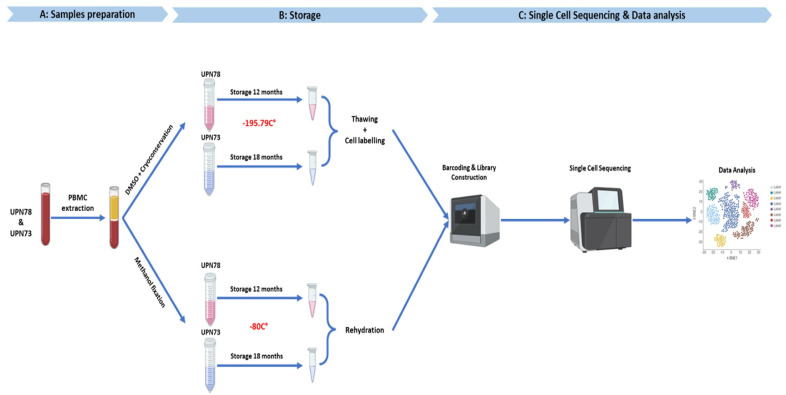
Experimental processing and sequencing of methanol-fixed and cryopreserved samples workflow. A: sample preparation, B: storage, C: single-cell sequencing and data analysis.

To initiate the thawing process, unfixed cells cryopreserved in liquid nitrogen were warmed to 37 °C. Subsequently, the cells were rinsed with RPMI 1640 medium to eliminate the freezing medium containing DMSO. In order to enhance the sample quality by enriching it with viable cells, a Dead Cell Removal Kit (MACs Miltenyi Biotec, Bergisch Gladbach, Germany) was used according to the manufacturer’s instructions to eliminate dead cells. A visual overview of the various steps involved in cryopreservation, methanol fixation, and rehydration is presented in Figure 1.

The processing and bioinformatic analysis of single-cell RNA-seq was carried out following the established protocols described in a previous study [19].

### 2.2. Single-Cell RNA-Seq Processing and Data Analysis

#### 2.2.1. Cell Labeling with Cell Hashing Antibodies

To initiate the cell labeling process, a 50 μL aliquot containing 1,000,000 cells suspended in a Cell Staining Buffer (CSB) with a viability of over 95% was prepared for the unfixed sample. The TotalSeq-A0251 (HTO1) and -A0252 (HTO2) antibody-oligo (Biolegend) were utilized for labeling. Each cell suspension received 1 μg of HTO for labeling. The two labeled samples were then incubated at 4 °C for 30 min. Subsequently, the cells underwent three consecutive washes with 3.5 mL of CSB followed by centrifugation at 400× *g* for 5 min at 5 °C. Finally, the cells were resuspended in PBS at a concentration of 1 × 10^6^ cells/mL.

#### 2.2.2. Single-Cell Library Generation and Sequencing

The single-cell RNA-seq technique was implemented following the guidelines outlined in two user manuals provided by 10x Genomics. For the fixed samples, we referred to the “Chromium Next GEM Single Cell 3′ Reagent Kits v3.1” (cat # CG000204 Rev B), while for unfixed samples, we utilized the “Chromium Next Gem Single Cell 3’ Reagent Kits v3.1 with Feature Barcoding technology for Cell Surface Protein” user guide (cat # CG000206 Rev D).

In summary, the samples were prepared by diluting them to a suitable concentration to recover 10.000 cells/sample. The single-cell suspension was then emulsified in Gel Beads in Emulsion (GEM) using the 10X Chromium Controller instrument. Subsequent steps included cell lysis, barcoded reverse transcription of RNA, and amplification. Following 5′ adapter ligation, we indexed the samples for sequencing. Finally, the sequencing of libraries was performed using the NextSeq 500/550 High Output Kit v2.5 (75 cycles, Illumina) in accordance with the manufacturer’s recommendations.

#### 2.2.3. Single-Cell RNA-Seq Data Processing

Sequencing data were processed using the 10X Cell Ranger pipeline and Seurat R package. Raw data were converted to FASTQ files with the CellRanger mkfastq function, and the reads were subsequently aligned to the human genome (GRCh 38) with CellRanger count. Seurat objects were created for the fixed samples based on the following filter criteria: a minimum of 3 cells, a minimum feature number of 200, and cell this <25% of mitochondrial genes. For the fixed sample, “log-normalization” of the Seurat object was performed for individual normalization. 

For processing multiplexed samples, the HTO data were handled using the “cite-seq count, v1.4.3” tool (https://hoohm.github.io/CITE-seq-Count/ (accessed on 10 May 2022) & https://zenodo.org/record/2590196 (accessed on 10 May 2022)). The outcomes were then read with the Seurat “Read10X” tool (https://satijalab.org/seurat/articles/hashing_vignette.html (accessed on 10 May 2022)). Only the common cell barcodes present in both the HTO data (from cite-seq count) and the mRNA data (from CellRanger) were retained. These shared barcodes represent the cells detected with CellRanger and for which an HTO was assigned. Subsequently, the HTO arrays were normalized using the Log-Ratio Centered (CLR) method, a recommendation from Seurat developers. To assign an HTO name to the cells, the “MULTIseqDemux” function was utilized.

#### 2.2.4. Dimension Reduction, Clustering, and Data Visualization

The top 2000 most highly variable features that exhibit high cell-to-cell variation from each sample were selected for data integration. Next, the top 15 principal components of the integrated data were selected for principal component analysis, UMAP analysis, and graph-based clustering to identify distinct subpopulations. Classification of PBMCs was inferred based on the annotation of cluster-specific genes, relying on the expression of well-known markers for immune cell types (marker-based classification). For a comprehensive exploration of the dataset, Loupe Cell Browser (v5.0) was used, allowing for an interactive analysis to identify significant genes, cell types, and substructures within the cell clusters [20].

### 2.3. Gene Ontology Analysis for Molecular Function

Gene Ontology analysis for molecular function was performed using the Panther version 16.0 database (http://pantherdb.org/ (accessed on 10 May 2022)).

### 2.4. Code Availability

All codes used in this study are available online at GitHub-SebastienNin/mw-Madaci2021 (https://github.com/SebastienNin/mw-Madaci2021 (accessed on 10 May 2022)).

Figure 1 summarizes the various steps involved, from cell preparation to the sequencing process.

## 3. Results

### 3.1. Quality Control before Sequencing the Fixed and Cryopreserved Samples

This study examines the efficacy of methanol fixation for the preservation of PBMCs at the single-cell level in two AML patients. We compared this method with DMSO cryopreservation on these samples for a period of one year or more. For this, we used the single-cell RNA sequencing approach. After the generation of GEMs and cDNA synthesis, quality control was performed using a fragment analyzer. This fragment analysis involves examining fluorescently labeled cDNA fragments, where expression peaks indicate cDNA fragment size. The main interest of this QC lies in the analysis of cDNA fragment sizes after the fragmentation step.

**Figure 2:**
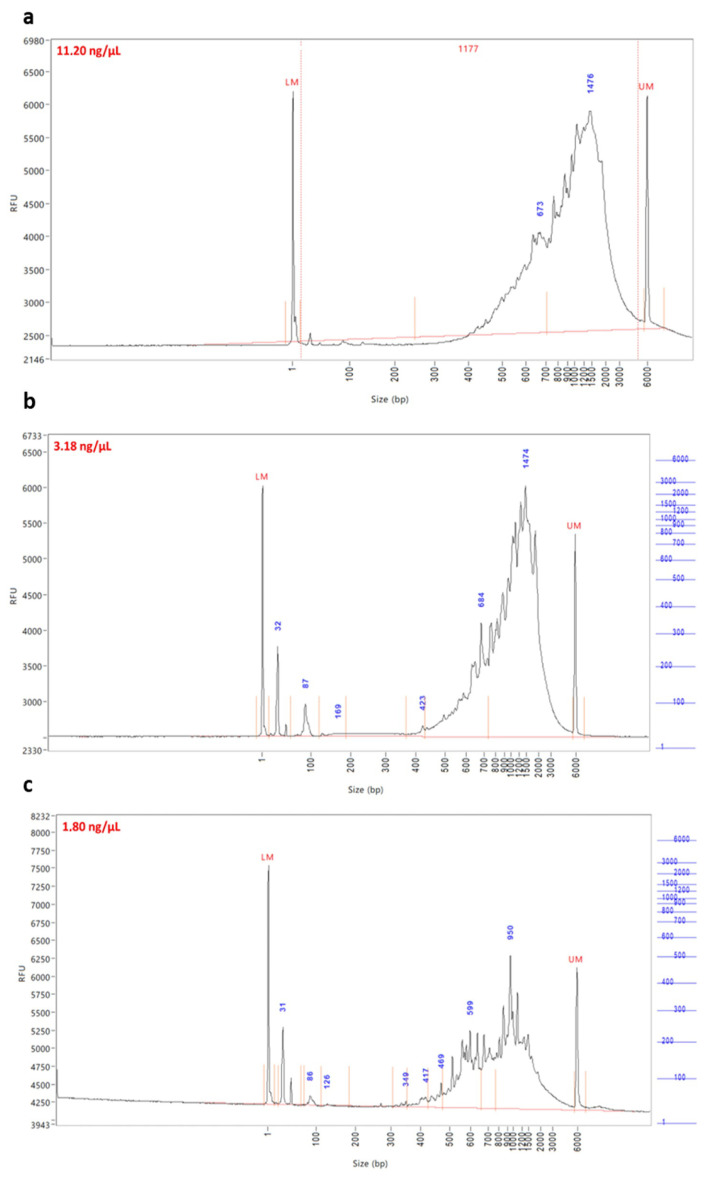
cDNA traces after quality control: (**a**) cDNA traces of unfixed UPN78/UPN73 multiplexed samples, (**b**) cDNA profile of UPN78-fixed sample for 12 months, (**c**) cDNA traces of UPN73-fixed sample for 18 Months. cDNA concentration for each condition is provided.

We observed cDNA fragments between 400 and 9000 base pairs (bp), confirming the presence of a cDNA library usable for DMSO cryopreserved UPN78 and UPN73 samples, showing a concentration of 11.20 ng/µL (Figure 2a). However, we noted material loss when the samples were fixed with methanol for 12 months (Figure 2b) and 18 months (Figure 2c), with concentrations of cDNA ranging from 3.18 ng/µL to 1.80 ng/µL, respectively.

### 3.2. Comparison of the Number of Transcripts and Genes Detected in Leukemia Cells in Methanol Fixation Compared with the Cryopreservation Method

After confirming the quality of the cDNA in both fixed and unfixed samples, we proceeded with RNA sequencing using 10 Genomics’ scRNA-seq technology. We individually sequenced approximately 10,000 cells for each sample. Following the application of filters during the post-sequencing quality control phase, we obtained a total of 5840 cells for the UPN78_DMSO sample and 10,096 cells for the UPN78 sample that had been fixed for 12 months. Additionally, we conducted sequencing for 4122 cells from the UPN73_DMSO sample and 10,781 cells from the UPN73 sample that had been fixed for 18 months.

**Figure 3:**
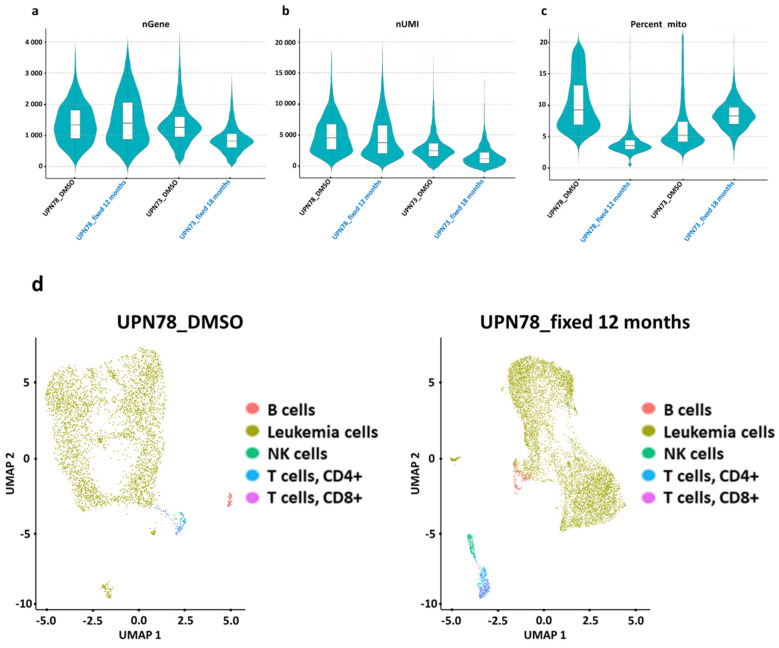

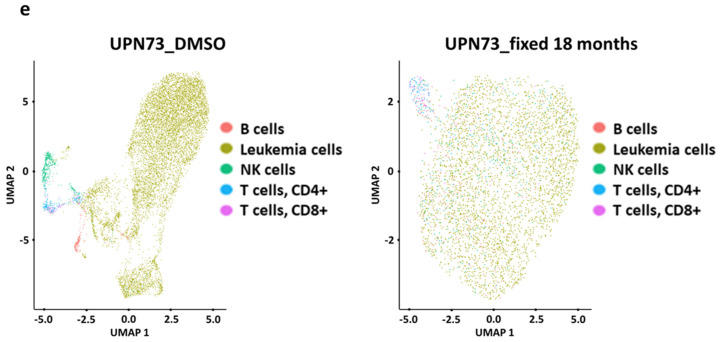
Preservation of single-cell RNA profiling and gene expression levels through fixation processing. (**a**–**c**) The figures display different quality control (QC) measures comparing methanol-fixed (blue) and cryopreserved (black) AML cells sequenced using single-cell RNA sequencing. (**a**) A violin plot visualizes the gene detection count, (**b**) the count of unique molecular identifiers (UMIs), and the percentage of mitochondrial genes (**c**) in each cell for the UPN78-fixed 12-month sample versus the UPN78-DMSO samples and the UPN73-fixed 18-month sample versus UPN73-DMSO samples. (**d**,**e**) UMAPs (uniform manifold approximation and projections) are used to visualize the distribution of various cell populations, encompassing B cells, NK cells, CD4+ T cells, CD8+ T cells, and leukemia cells in UPN78 (**d**), both cryopreserved (**left**) and methanol-fixed for 12 months (**right**), as well as UPN73 (**e**), cryopreserved (**left**), and methanol-fixed for 18 months (**right**). In this visualization, each cell type is assigned a unique color to aid in their identification and comparison.

In the UPN78_DMSO sample (Figure 3a), we observed a median of 1567 genes per cell (first quartile: 1108; third quartile: 2068). Similarly, the UPN78_fixed sample showed a median of 1546 genes per cell (first quartile: 1019; third quartile: 2219). For the UPN73 sample (Figure 3a), we detected a median of 1329 genes per cell in the UPN73_DMSO sample (first quartile: 1049; third quartile: 1644), whereas the UPN73_fixed sample exhibited a median of 800 genes per cell (first quartile: 598; third quartile: 1067).

In terms of the number of captured unique molecular identifiers (UMIs), we observed a median of 4339 UMIs for the UPN78_DMSO sample and 3754 UMIs (first quartile: 2637; third quartile: 6383) for the UPN78_fixed 12-month sample (first quartile: 2206; third quartile: 6269). Conversely, the UPN73_DMSO sample showed a median of 3185 UMIs (first quartile: 2329; third quartile: 4296), while the UPN73_fixed 18-month sample exhibited 1427 UMIs (first quartile: 1021; third quartile: 1985) (Figure 3b).

Finally, we conducted a comparison of the proportion of mitochondrial genes between the two experimental conditions. In the UPN78_fixed sample, we observed a median of 3% mitochondrial genes (first quartile: 2.3%; third quartile: 3.7%), whereas the UPN78_DMSO sample exhibited a median of 10% mitochondrial genes (first quartile: 6.8%; third quartile: 15%). For the UPN73 samples, the UPN73_fixed condition had a median of 9% mitochondrial genes (first quartile: 7%; third quartile: 10%), while the unfixed condition, UPN73_DMSO, had a median of 6% mitochondrial genes (first quartile: 5%; third quartile: 9%) (Figure 3c).

After conducting quality assessments across various factors, including gene expression, UMIs, and mitochondrial gene ratios, it was noted that prolonged methanol fixation of AML PBMCs for over 12 months leads to material degradation.

After quality control, we investigated the impact of methanol fixation on the distribution of normal peripheral blood mononuclear cells (PBMCs) in the analyzed samples. Specifically, we compared the percentages of B lymphocytes, CD4+ and CD8+ T lymphocytes, and NK cells to assess the effect of fixation.

In the fixed UPN78 sample compared with the cryopreserved sample, we observed 2.60% vs. 1.85% B cells and 3.12% vs. 0.66% NK cells. For T cells, the percentages of fixed vs. cryopreserved CD4+ cells were 3.97% vs. 1.34%, and for CD8+ cells, they were 1.78% vs. 0.76% (Figure 3d). Similarly, in the fixed UPN73 sample compared with the cryopreserved sample, we observed 4.66% vs. 1.95% B cells, 7.38% vs. 4.18% NK cells, 3.21% vs. 2.12% CD4+ T cells, and 1.99% vs. 1.09% CD8+ T cells (Figure 3e). The results of this analysis show significant differences in the cell populations weakly present in UPN78 and UPN73. The percentages of the different cell populations defined with a flow cytometry analysis better fitted with the fixed condition than in the cryopreservation one. This suggests that fixation did not result in significant changes in normal cell populations. In addition, we carried out a similar comparison focusing on AML cells specifically, identifying them based on RNA expression corresponding to the leukemia phenotype as detected with flow cytometry (see Table 1).

**Figure 4:**
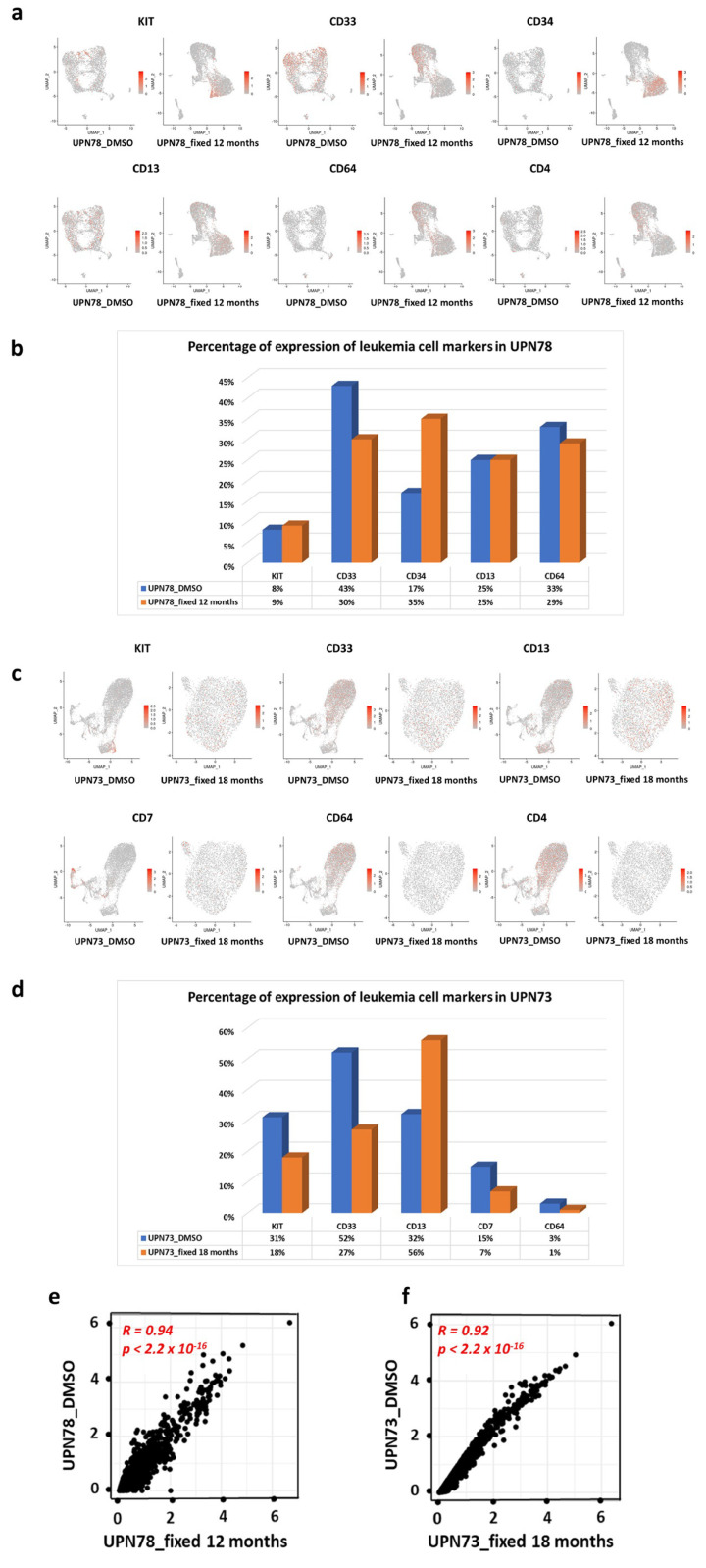
The expression of leukemia cell-specific markers remains unaffected by methanol fixation. (**a**) The UMAP visualization depicts the contrast in the expression of leukemia cell markers (KIT, CD33, CD34, CD13, CD64, and CD4) between UPN78 cryopreserved in DMSO and UPN78 methanol-fixed for 12 months. (**b**) The histogram presents the percentage expression of these markers in UPN78 under DMSO cryopreservation versus methanol fixation for 12 months. (**c**) The UMAP representation highlights the comparison of leukemia cell marker expression (KIT, CD33, CD13, CD7, and CD64) between UPN73 cryopreserved and UPN73 fixed for 18 months. (**d**) Additionally, a histogram is provided to demonstrate the percentage expression of these markers in UPN73 under cryopreserved conditions and fixed for 18 months. (**e**,**f**) The Spearman correlation analysis reveals the correlation of average gene expression between UPN78 cryopreserved and UPN78 fixed for 12 months (**e**), and cryopreserved UPN73 compared to the fixation condition for 18 months (**f**).

Initially, we examined the expression of leukemic markers, including KIT (CD117), CD33, CD34, CD13, and CD64, in UPN78 cells preserved in DMSO cryopreservation compared with those fixed with methanol. Our analysis, represented in Figure 4a (UMAP plot) and Figure 4b (cell percentage plot), indicated a concordance between the fixed and unfixed conditions, with consistent marker expression. Subsequently, we investigated the impact of long-term fixation (beyond one year) on the expression of leukemic markers in UPN73. We specifically examined the expression of KIT (CD117), CD33, CD13, CD7, CD64, and CD4 in both the fixed and non-fixed UPN73 samples (Figure 4c). The results revealed that fixing the cells for an extended period led to a decrease in the expression of certain leukemic markers (Figure 4d). These findings demonstrated a correlation in marker expression between leukemic cells preserved with cryopreservation or fixed with methanol for up to one year. However, it is noteworthy that cell fixation moderately loses its effectiveness when maintained for an extended duration.

### 3.3. Gene Expression Correlation between Methanol Fixation and Cryopreservation

For this initial analysis, we examined the correlation between the average expression of all genes in cryopreserved and methanol-fixed samples. As depicted in Figure 4e,f, we observed a strong correlation between the gene expression levels of the cryopreserved and fixed UPN78 and UPN73 samples, with correlation indices of R = 0.94 (*p <* 2.2 × 10^−16^) and R = 0.92 (*p <* 2.2 × 10^−16^), respectively. These findings indicate that methanol fixation does not significantly impact the overall cell transcriptome compared with DMSO cryopreservation. Based on this preliminary analysis, we can conclude that the overall gene expression profiles remain highly consistent between the two preservation methods.

### 3.4. Methanol Fixation Cellular Heterogeneity after Single-Cell RNA Sequencing Compared with Cryopreservation

**Figure 5:**
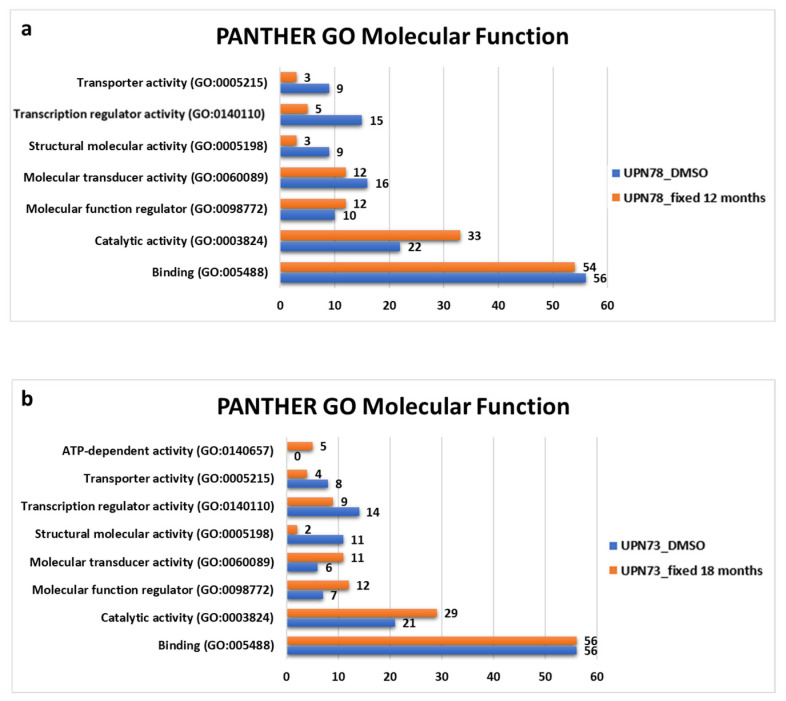
Preservation of cellular heterogeneity maintained with methanol fixation in single-cell sequencing. (**a**,**b**) Bar chart illustrating the Panther gene ontology molecular function analysis of the top 200 significant genes in each condition of UPN78 (**a**) and UPN73 (**b**).

To evaluate the efficacy of methanol fixation on leukemia cells, our study focused on transcriptome analysis and aimed to understand its impact on gene expression models. To this end, we meticulously selected the top 200 genes in each condition and then examined their molecular functions using the Gene Ontology tool. By comparing the UPN78 samples that were fixed or cryopreserved (Figure 5a), we identified seven molecular functions with disparities, especially in the number of genes involved in the transport activity, transcription regulator activity, and structural molecular activity, which exhibited a decrease in fixed state. Similarly, when evaluating the UPN73 samples that were fixed or cryopreserved, we identified eight shared molecular functions, with some variances between the two conditions (Figure 5b). These results highlight a certain level of coherence between the two preservation methods, with some distinctions emerging in terms of transcriptomic analysis conducted at the cellular level of leukemia cells. This finding affirms the utility of methanol fixation as a viable strategy to preserve the transcriptome, confirming its suitability for further investigation in leukemia research.

## 4. Discussion

Our comparative analysis demonstrates that leukemic samples fixed with methanol maintained a comparable gene count to cryopreservation for up to 12 months of fixation. However, with prolonged methanol fixation, a notable decrease in the gene count was evident, alongside a similar trend in UMI count. Conversely, there was a contrasting increase observed in mitochondrial genes. Overall, these findings indicate that extended methanol fixation leads to material degradation. In profiling cellular populations within AML samples, T/B lymphocytes and NK cells, being infrequent due to predominant leukemic cell circulation, displayed variable percentages between methanol fixation and cryopreservation. Despite this, the percentages of cells across both methods aligned within a similar range when compared with flow cytometry data. Moreover, cellular populations identified with flow cytometry, predominantly reliant on surface protein detection, differed somewhat from RNA expression identification. The expression of leukemia cell markers similarly showcased considerable variation with extended methanol fixation versus cryopreservation. Furthermore, distinctions were noted between the two methods concerning the identification of molecular function pathways, particularly with less abundant gene functions (e.g., transporter activity), while more consistent results were observed for more prevalent molecular functions (e.g., binding). Although the high-resolution observation reveals notable methodological differences, a comprehensive statistical analysis of leukemia-specific markers demonstrates a strong correlation between methanol and cryopreservation techniques (R = 0.94 and 0.92 for UPN78 and UPN73, respectively, according to the Spearman analysis).

The outcomes align with the findings presented in Chen et al.’s 2018 study [13], in which they scrutinized the transcriptome of fixed PBMCs using single-cell RNA sequencing. Their research encompassed normal PBMCs and cell lines, while our study focused on just two normal PBMC samples, emphasizing primary cells. It is crucial to note that our analysis involved primary normal cells and used methanol fixation durations spanning 3 h to 3 weeks, potentially contributing to material degradation. Consequently, our study exhibited less variability compared with theirs, revealing correlation scores ranging from 0.95 to 0.98. Moreover, the application of RAMAN spectroscopy to analyze methanol fixation illustrated reduced discriminatory capacity between normal and cancer cells, presenting a significant overlap in populations not witnessed with paraformaldehyde fixation during fresh sample analysis [21].

These initial findings highlight the effectiveness of the fixation method in preserving cells over a relatively short duration. Nevertheless, it is crucial to acknowledge certain limitations within this study, including the small sample size and the utilization of a single treatment point for each sample.

## 5. Conclusions

Methanol fixation proves to be a practical approach for the preservation of biological species, particularly beneficial when conventional cryopreservation methods pose challenges for single-cell analysis while preserving the integrity of genomics, transcriptomics, and molecular components. Its simplicity of implementation makes it an appropriate choice for ongoing single-cell investigations, especially in the field of hematology, emphasizing the discovery of the subtleties of leukemia blasts.

Although the methanol fixation technique may produce variable results in quantifying certain cell populations and expressing certain functional pathways, it remains applicable to the analysis of leukemia blasts fixed over a short period, which could facilitate its integration into current clinical practice.

## Figures and Tables

**Table 1:** Clinical characteristics of the samples.

Sample	UPN78	UPN73
Sex	M	M
Age	23	50
Nature of samples	Blood	Blood
% of blasts in blood	79%	86%
Cytology	AML4	AML2
Prognostic group	Favorable	Intermediate
Phenotype	CD117, CD34, CD33, CD13 (myeloid blasts 20%), CD64, CD33 (monocytic blasts 70%)	CD117, CD33, CD13, CD7, CD64, CD4.
Cytogenetic	46XY, inv (16)	Normal karyotype
Genetic abnormalities	FLT3-TKD, WT1	DNMT3A, FLT3-ITD, FLT3-TKD, KIT, NPM1

UPN: unique patient number; M: male; AML: acute myeloid leukemia; CD: cluster of differentiation; inv: inversion; FLT3: Fms-like receptor tyrosine kinase class III; ITD: internal tandem duplication; TKD: tyrosine kinase domain; WT1: Wilms tumor 1; DNMT3A: DNA methyltransferase 3 alpha; KIT: kit proto-oncogene, receptor tyrosine kinase; NPM1: Nucleophosmin 1.

## Data Availability

The data presented in this study are available in the NCBI Gene Expression Omnibus (GEO) public database (GSE237239).
